# Trends (2014–2018) in the healthiness of packaged food purchases of Australian consumers before and after the introduction of voluntary Health Star Rating nutrition labels

**DOI:** 10.1017/S1368980024000892

**Published:** 2024-04-11

**Authors:** Satheesh Seenivasan, Anish Nagpal, Dominic Thomas, Gary Sacks

**Affiliations:** 1 Monash Business School, Monash University, Clayton, VIC 3168, Australia; 2 Department of Management and Marketing, University of Melbourne, Parkville, Australia; 3 Global Centre for Preventive Health and Nutrition, Deakin University, Burwood, Australia

**Keywords:** Health Star Ratings, Nutrition summary indicators, Trends in healthiness, Nutrition labels

## Abstract

**Objective:**

To evaluate the trends in the healthiness of packaged food purchases of Australian consumers before and after the introduction of the Health Star Rating (HSR) nutrition labels.

**Design:**

Panel data analysis and difference-in-differences analysis.

**Setting:**

The Australian Government endorsed HSR nutrition labels for voluntary implementation on packaged foods in June 2014. We analyse the packaged food purchases of households across all major supermarkets before (January 2014 to June 2014) and after (June 2014–Dec 2018) the introduction of HSR.

**Participants:**

6284 members of a panel of households across Australia reporting their grocery purchases to a market research company (Nielsen Homescan panel).

**Results:**

The healthiness of household food purchases exhibited a U-shaped trend – decreasing from 2014 to 2017, and then increasing from 2018, corresponding to the time when a higher proportion of products were HSR-labelled. Households that purchased a higher proportion of HSR-labelled products had healthier household purchases overall. Further, the healthiness of households’ category-specific food purchases was positively associated with the proportion of HSR-labelled products in categories where HSR was adopted, relative to control categories where HSR was not adopted.

**Conclusions:**

In Australia, once a substantial number of packaged food products adopted the voluntary HSR summary indicator, we observed an increasing trend in the healthiness of household food purchases. Widespread adoption of a nutrition summary indicator, such as HSR, on packaged food is likely to be beneficial for population health.

Nutrition summary indicators aim to help consumers select healthier foods by providing a simple and easily understandable measure of the overall nutrition quality of food products^(1–4)^. Several different formats of nutrition summary indicators have been adopted worldwide, such as the Nutri-Score system in parts of Europe and the Health Star Rating (HSR) scheme in Australia and New Zealand^(5,6)^. In 2014, the Australian Government endorsed the HSR labelling scheme as a voluntary front-of-pack nutrition summary indicator for packaged food products (foods that are sealed in a package such as breakfast cereals and soft drinks)^(6)^.In the HSR scheme, each food product is assigned a rating from ½ star to 5 stars in increments of 1/2 stars. Higher ratings (i.e. more stars) indicate a healthier product. HSR is calculated based on the product’s total energy, saturated fat, sugar, sodium, fibre, protein, fruit, vegetable, nut and legume content per 100 g or 100 ml of the product^(6)^. Food industry began voluntary implementation of HSR in 2014, with less than 10 % of food and beverage products displaying HSR labels in 2015, increasing to 43 % of products by 2023, and the extent of implementation varied both across categories and within categories over time^(7,8)^. However, whether nutrition summary indicators, such as HSR, lead to healthier purchases remains an empirical question.

Several studies have examined the effects of nutrition summary indicators on consumer purchases. However, a major limitation of most of these studies is that they are lab-based or survey-based studies with a focus on stated rather than actual purchase behaviour. The very few studies on nutrition summary indicators that have examined actual consumer food purchases have been limited to analysing purchases in a small number of product categories in one or a few grocery retailers for a limited time period^(9–13)^. For example, Dubois and colleagues used sales data on four product categories in three grocery retailers for 10 weeks and showed that the Nutri-Score scheme was associated with an increase in the sales of the healthiest products within a category, but had no impact on the sales of other products in the category^(9)^. The estimated effect sizes in their study were found to be 17 times smaller than those found in laboratory studies, emphasising the importance of studying real-life purchase behaviour^(9)^. An exception is the study by Bablani and colleagues which analysed the effect of HSR labels on packaged food products purchases of 2500 households in forty-three categories in New Zealand^(12)^. However, the study focused on individual products, which does not provide insights into the effects of labelling on the healthiness of purchases at the overall household level. Importantly, studies that focus on few categories in few stores are unlikely to be able to account sufficiently for the implications of switching of purchases across product categories (e.g. from soft drinks to fruit juices) and across different store types (e.g. fresh foods from one store and packaged foods from another store).

In this study, we evaluated the trends (2014–2018) in the healthiness of households’ food purchases across all packaged food categories across all grocery stores before and after the introduction of the HSR scheme. We also analysed the association between the purchases of HSR-labelled products and the healthiness of products purchased and the trends in the healthiness of category-specific purchases.

## Methods

### Data

The primary source of data for this study was the Homescan grocery panel data from Nielsen. This nationally representative panel dataset records the grocery purchases of Australian households across all categories and grocery stores. The dataset covers product information (product or Universal Product Code (UPC), brand, size), transaction information (date, store, quantity, price paid) and household information (household size, location, primary shopper’s age, income level, life stage and affluence).

The panel dataset used in the study covers transactions made by 13 339 households across Australia during the 5-year period: January 2014 to December 2018, which includes 6 months of data before the endorsement of HSR (pre-HSR period) and 54 months of data after the endorsement of HSR (post-HSR period). However, not all of the panel households were available throughout the data period. To keep the panel composition consistent, we included only those households that were available for the entire data period in our main analysis, resulting in a final sample of 6284 households. The demographic profiles of sample households are summarised in online supplementary material, Supplemental Table 1.

We analysed the changes in the healthiness of household purchases across all categories of packaged food, excluding a limited number of categories (such as baby foods and vitamins) that are ineligible for HSR^(6)^. As HSR is not applicable to non-packaged fresh food categories like fruits and vegetables, we focused only on packaged food categories (e.g. breakfast cereals, sauces, breads) for our main analysis. Our analysis covered 114 packaged food categories, accounting for 78·6 % of total household food spending and 96 % of total household packaged food spending.

We obtained data on the nutrition content of packaged food products from the FoodSwitch ® database provided by The George Institute for Global Health^(7)^. This dataset includes annual data of the nutrient levels (energy, fat, saturated fat, sugar, Na), HSR (rating and whether HSR was displayed) and serving sizes of over 50 000 packaged food products sold in Australian supermarkets. For packaged food products that did not display HSR, the FoodSwitch database provided an estimated HSR based on the product’s nutrition content. The products available in the FoodSwitch database covered 66 % of sales (by dollar value) of households in the Nielsen panel data. For packaged products purchased by Nielsen households that were not covered in the FoodSwitch database, a research assistant manually collected their nutrition content and HSR from manufacturer and retailer websites. The data collected manually accounted for 29 % of the dollar sales of Nielsen households. For a small number of products for which nutrition information was not available (accounting for 5 % of total dollar sales of Nielsen households), we imputed the nutrition content using the average nutrition content of all products in the same category (e.g. average energy of all breakfast cereals was used to impute the energy of a breakfast cereal product with missing data).

### Outcome measures

The primary outcome measure was the healthiness of quarterly household packaged food purchases (hereafter referred to as food baskets). For the main analysis, the household food baskets comprised of 114 packaged food categories covered in the study. The healthiness of the food baskets was measured using the HSR of the food basket (food basket HSR, described below) and the total energy (kJ) in the food basket for each quarter for each household.

Food basket HSR was calculated as the share weighted average HSR of individual products (UPC) in households’ food baskets. The share weight of a UPC is its dollar share in households’ food basket (i.e. total dollars spent on the UPC/total food basket spending of the household). So, if a household *h* purchases *n* UPC in quarter *t* and the HSR of each UPC *i* is denoted as *HSR*_*i*_, then the HSR rating for the household’s food basket is calculated as,

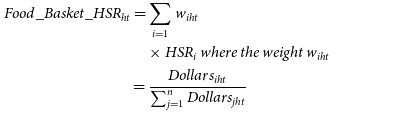




where 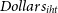 is the total dollars spent by household *h* in quarter *t* on UPC *i*. As a higher level of HSR implies a healthier product, a higher value of the food basket HSR indicates a healthier basket. We used HSR (actual or estimated) of all products (not just those that displayed HSR) purchased by households in calculating the food basket HSR, to ensure that it reflects the healthiness of overall household purchases and is not biased by the proportion or the type of products choosing to display HSR labels (e.g. higher tendency of healthier products to display HSR labels^(7)^). For the category-wise analyses, we computed the category-specific HSR separately for each of the 114 studied categories.

As an alternate measure of the healthiness of food baskets, we calculated the total energy (kJ) purchased by households every quarter. Total energy purchased is commonly used as a summative measure of healthiness of purchases in the existing literature^(14)^. To compute this measure, we first calculated the energy from each product purchased by a household as the product of energy per 100 units (g or ml) of the product (from the nutrition information panel), size of the product and the total quantity of the product purchased by the household in a quarter. We then calculated the total energy purchased by a household in a quarter as the sum of energy from all the packaged food products purchased by the household in that quarter. To account for differences in the total energy purchased across households due to differences in household size and composition, we normalised the total energy purchased by each household by diving it by the average energy purchased by the household in the first 6 months of data (i.e. pre-HSR) period. While a higher food basket HSR indicates a healthier basket, higher total energy in the food basket indicates a less healthy basket.

### Statistical analysis

As HSR is a voluntary scheme, the trend in the adoption of HSR labels by products was not linear. We used two different models to account for potential non-linear trends in food basket healthiness. First, we estimated the quarterly changes in food basket healthiness (i.e. food basket HSR and total energy in the food basket) within households using regression models with household and time (i.e. quarter) fixed effects (equation 1).
(1)

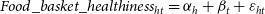




In this quarter-wise fixed effects model (equation 1), 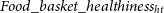 is the healthiness of packaged food purchases of household *h* in quarter *t*. Household fixed effects ( 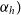 control for the differences in basket healthiness across households due to differences in time-invariant household characteristics. Quarter fixed effects ( 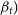 estimate the average changes in food basket healthiness within households relative to the first quarter. In addition to the full sample, we also analysed the trends in the basket HSR of households by sub-groups based on age, gender and household income, and the results of this analysis are presented in online supplementary material, Supplemental Appendix 2.

Second, we estimated a quadratic trend model with household fixed effects and quadratic time trends (equation 2).
(2)

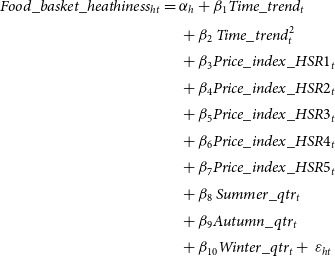




In the quadratic trend model, 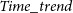 refers to the quarterly time trend common across all households. In this model, we also controlled for potential differential trends in prices of products with different HSR levels by including five quarterly price indices ( 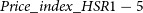 ), one each for products with HSR scores within the following ranges: 0·5–1, 1·5–2, 2·5–3, 3·5–4 and 4·5–5. Price indices were calculated as the share weighted average price per serve of products with HSR scores within the above ranges, where the weights are the dollar shares of a product in the total dollar sales of all products in that HSR range. To control for seasonality, we included categorical variables to represent quarters that correspond to summer, winter and autumn seasons (relative to spring).

To analyse whether the observed trends in the healthiness of households’ food baskets are consistent with households’ purchasing healthier foods after HSR labelling, we conducted two different analyses. First, we tested whether the healthiness of food baskets was higher for households that purchased more HSR-labelled products by estimating the association between households’ food basket healthiness and the proportion of HSR-labelled products in their food basket (equation 3).
(3)

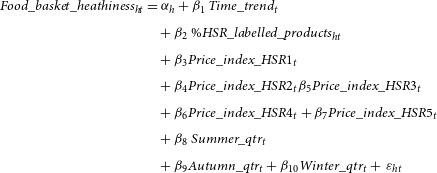




where 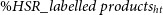 is the proportion of products in households’ basket that actually displayed HSR. We obtained the data on whether or not a product actually displayed HSR from the FoodSwitch database.

Lastly, we tested whether household purchases were healthier in categories where more products were HSR-labelled, using a multi-level fixed effects difference-in-differences type regression model. Specifically, we estimated the changes in the healthiness of households’ category-specific purchases before and after the adoption of HSR labels in treatment categories relative to control categories where HSR labels were not adopted, and how the changes in healthiness varied with the proportion of products in the treatment categories that were HSR-labelled (equation 4).
(4)

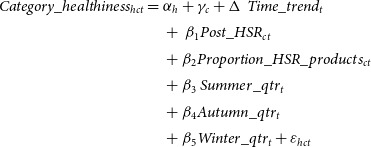




where 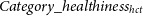 is the HSR of category-specific purchases of household *h* in category *c* in quarter *t*. 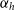 and 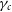 are household and category-specific fixed effects, and 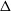 captures the effect of time trend. 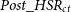 is a categorical variable which indicates whether HSR was adopted by products in a category *c* in quarter *t*. Conditional on 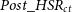 being 1, the proportion of HSR-labelled products in category *c* in time *t* ( 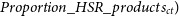 will be greater than 0. The coefficient of this term captures how the effect of HSR adoption on the healthiness of household purchases varied depending on the proportion of products in the category that were HSR-labelled.

We used household purchases in all studied 114 categories for this analysis. As all households may not purchase all categories every quarter, the dataset was an unbalanced panel dataset. The control categories for this analysis included those in which HSR labels were not adopted during the study period (e.g. ethnic foods, mixes and batters). Additionally, categories in which HSR adoption started in later years (e.g. HSR labels were first observed on products in the canned meals and carbonated fruit juice categories from 2017, and from 2016 in eggs) served as additional control groups for categories in which HSR adoption started earlier (e.g. HSR labels were first observed in breakfast cereals in 2014) for the intervening years (i.e. 2015 and 2016). More details of the model specifications are provided in online supplementary material, Supplemental Appendix 1.

### Robustness checks

We conducted multiple tests to assess the robustness of our results to alternate measures of basket healthiness and to the household sample used in the analysis. To assess whether our results were robust to alternate measures of food basket healthiness, we calculated basket HSR using share of energy (total energy from a UPC purchased/total energy in the food basket) as the weight, instead of share of dollar spending. Results of this analysis are shown in column 3 of online supplementary material, Supplemental Tables 3–6.

Second, our main analysis excluded fresh foods, like fruits and vegetables. For robustness, we also calculated basket HSR including fresh food purchases of households (i.e. covering all food purchases in the dataset). We assigned a HSR of 5 to all fresh food products for this analysis, consistent with guidance provided by the Australian Government^(6)^. The results of this analysis are discussed in online supplementary material, Supplemental Appendix 3.

Third, we assessed the robustness of our results to the sample used in the main analysis by analysing an extended sample that covered all households that were available in the pre-HSR period and had at least 2 years of data. The resulting unbalanced panel data covered 7996 households. We re-estimated all our models with this extended sample (results are presented in online supplementary material, Supplemental Tables 5–6).

### Role of the funding source

None.

### Findings from the empirical analyses

#### Trends in HSR adoption and purchase over time

**Fig. 1:**
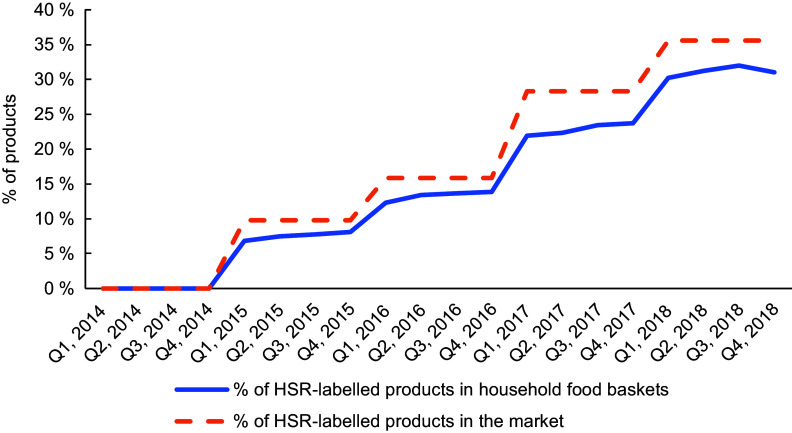
Trends in the proportion of Health Star Rating (HSR)-labelled products in the market and the average proportion of HSR-labelled products in household food baskets over time. The proportion of HSR-labelled products in the market and household baskets is measured for each quarter (Q1 to Q4) for each study year (2014–2018)

We first present the longitudinal trends in the proportion of HSR-labelled products in the market (number of products with HSR label/number of products in the FoodSwitch database) and the average proportion of HSR-labelled products in household food baskets (i.e. number of products with HSR label/total number of products in the basket) every quarter in Fig. 1.

The proportion of products that had adopted HSR increased from 0 % in 2014 to 6·85 % at the start of 2015 and reached 35 % in 2018, exhibiting a monotonic but non-linear trend. The proportion of HSR-labelled products in household food baskets followed a similar trend, reaching a level of 28·5 % in the last quarter of 2018.

#### Trends in food basket healthiness

**Fig. 2:**
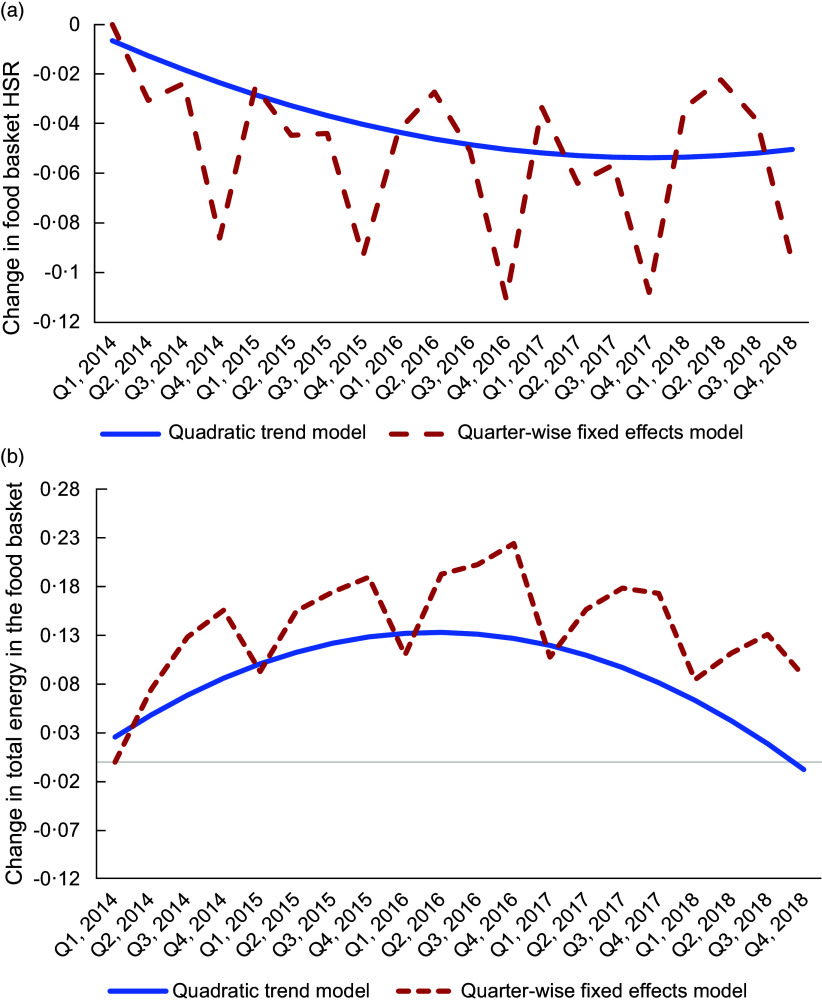
(a) Trends in the food basket Health Star Rating (HSR). Estimated changes in the food basket HSR (relative to the first quarter) from the quarter-wise fixed effects and quadratic trend models. HSR was endorsed by the Australian Government for voluntary adoption in Q2, 2014. (b) Trends in the total energy in the food basket. Estimated changes in the total energy in the food basket (relative to the first quarter) from the quarter-wise fixed effects and quadratic trend models. HSR was endorsed by the Australia Government for voluntary adoption in Q2, 2014.

The average food basket HSR across the studied households was 2·6, with a sd of 0·3. We first present the estimated trends in the two measures of household food basket healthiness (food basket HSR and total energy in the food basket) in Fig. 2(a) and (b) (corresponding regression estimates are presented in online supplementary material, Supplemental Tables 3 and 4).

Trends (Fig. 2(a)) show that the food basket HSR exhibited seasonality – it was lower in the last quarter (i.e. Q4) of every year (by 3·74 % on average across studied years). More importantly, food basket HSR exhibited a U-shaped trend – it decreased in the first 4 years (2014–2017) and then increased in 2018. Despite the reversal, the overall food basket HSR was slightly lower in 2018 compared with 2014. Specifically, the food basket HSR was lower by 0·4 % in Q4, 2018 compared with Q4, 2014.

Total energy in the food basket (Fig. 2(b)) exhibited a corresponding inverted U-shaped trend. Specifically, the total energy purchased increased in the first 3 years (2014–2016) of the study period and was 22·46 % higher in Q4, 2016 compared with Q1, 2014. However, the trend reversed in the last 2 years of the study period (2017–2018), and the total energy in the food basket in the last quarter (Q4) of 2018 was lower than that of Q4, 2016 by 13·7 %.

The non-linear trends in the food basket HSR and total energy are further supported by the estimates from the quadratic trend model (presented in online supplementary material, Supplemental Table 4). The results of this model illustrated in Fig. 2(a) and (b) show a U-shaped trend for the food basket HSR, and an inverted U trend for the total energy in the food basket. Estimates imply that the reversal in the trend for food basket HSR occurred in the fourth quarter of 2017.

We also analysed the trends in the food basket HSR of households by sub-groups based on age (<44 years, 45–54 years, >55 years), gender (male/female) and household income (<$40 000, $40–$80 000, $80–$140 000, >$140 000). Analysis of sub-groups revealed similar U-shaped trend for the food basket HSR and an inverted U-shaped trend for the total energy in the food basket for all sub-groups and minimal differences across sub-groups (results discussed in online supplementary material, Supplemental Appendix 2).

In summary, the healthiness of household food baskets exhibited a U-shaped trend – it decreased in the first 4 years (2014–2017), corresponding to a time when fewer products in the market had adopted HSR, and then exhibited an increasing trend in 2018, corresponding to a time when more products (35·6 % in 2018) had adopted HSR. Robustness checks conducted with alternate measures for food basket healthiness, inclusion of fresh foods in the calculation of food basket healthiness and an extended sample of households provided consistent results as the main analysis (results in online supplementary material, Supplemental Tables 5–7).

#### The association between food basket healthiness and purchase of products with Health Star Rating

We found a positive association between the purchases of HSR-labelled products by households and their food basket healthiness. As shown in Table 1, the proportion of HSR-labelled products in the basket was positively associated (*β* = 0·398, *P* < 0·001) with food basket HSR and negatively associated with the total energy in the food basket (*β* = –0·194, *P* < 0·001). Our estimates imply that, across the range of HSR implementation observed in the study, a 1 % increase in HSR-labelled products in the food basket was associated with a 0·15 % higher basket healthiness and a 0·2 % lower total energy consumed.

**Table 1:** Relationship between food basket healthiness and the proportion of Health Star Rating (HSR)-labelled products in the food basket

	Basket HSR		Total energy (KJ) in the basket	
	Estimates	se	Estimates	se
Time trend	−0·009***	0·0003	−0·001	0·001
Proportion of HSR-labelled products in the food basket	0·398***	0·008	−0·194***	0·023
Price_index_HSR_1	−0·130***	0·040	−0·865***	0·109
Price_index_HSR_2	0·074***	0·016	−0·407***	0·044
Price_index_HSR_3	−0·098	0·093	1·809***	0·252
Price_index_HSR_4	0·154**	0·048	0·023	0·131
Price_index_HSR_5	0·085***	0·017	−0·140**	0·046
Summer	0·056***	0·003	−0·035***	0·008
Winter	0·055***	0·002	−0·027***	0·005
Autumn	0·048***	0·002	−0·035***	0·006
R^2^	0·626		0·564	

Household specific fixed effects are not shown in the above table.

** *P* < 0·01.

*** *P* < 0·001.

There was also a significant negative (positive) trend in the food basket HSR (total energy in the food basket) during the study period. Taken together, our estimates (based on Q4, 2018) indicated that the basket healthiness was higher (i.e. the positive effect of HSR-labelled products in the basket exceeded the negative trend) when 45·5 % or more of products in household food baskets were HSR-labelled. In our sample, the proportion of HSR-labelled products in the basket was higher than this threshold (45·5 %) for 12·5 % of households.

As the basket healthiness measure used in the study is based on HSR rating (actual or estimated) of all products purchased by households, the construction of the outcome variables used in this analysis (basket HSR and total energy in the basket) is not influenced by the proportion and the characteristics of products choosing to display HSR labels. If HSR labelling did not lead to improvement in the healthiness of household purchases (i.e. basket healthiness), then there would not be any significant positive association between the basket healthiness measures (which would remain unchanged or exhibit a declining trend as indicated by our analyses) and the proportion of HSR-labelled products in the basket (which would exhibit an increasing trend as more products displayed HSR label over time). The observed positive association between the proportion of HSR-labelled products in the basket and the basket healthiness on the other hand was consistent with households purchasing healthier products after HSR labelling.

#### Category-wise analysis

Results from our category-wise analysis (shown in Table 2) indicated a negative trend in the healthiness of category purchases of households and lower healthiness in the period following the introduction of HSR labels. However, the effect of proportion of HSR-labelled products in the category on the healthiness of category-specific purchases was positive and significant. Specifically, our estimates (based on Q4, 2018) indicated that when 52·3 % of products in the category were HSR-labelled, there was a net increase in the healthiness of category-specific purchases of households. Results from all robustness checks were consistent with our main findings using dollar share weighted basket HSR.

**Table 2:** Relationship between category-specific Health Star Rating (HSR) and the proportion of HSR-labelled products in the category

	Category-specific HSR	
	Estimates	se
Time trend	−0·003***	0·0001
Post_HSR	−0·012***	0·001
Proportion of HSR-labelled products in the category	0·140***	0·004
Summer	0·008***	0·001
Winter	0·009***	0·001
Autumn	0·007***	0·001
R^2^	0·788	

Household specific fixed effects are not shown in the above table.

*** *P* < 0·001.

## Discussion

In this study, we investigated the changes in the healthiness of household quarterly food baskets (i.e. total packaged food purchases) before and after the introduction of HSR nutrition labels in Australia. We used actual grocery purchases of a nationally representative panel of Australian households across all packaged food categories and supermarkets over a period of 5 years (2014–2018). The study found a U-shaped trend in the healthiness of household food baskets – decreasing from 2014 to 2017 and then increasing in 2018. We further found that food basket healthiness was higher for households that purchased a higher proportion of HSR-labelled products. In addition, there was a positive association between the healthiness of category-specific purchases of households and the proportion of HSR-labelled products in the category. Taken together, these findings indicate that as more packaged products adopted HSR, the healthiness of household packaged food purchases increased. However, our results also showed an overall negative trend in the healthiness of food purchases since 2014, which is consistent with observed trends in key indicators of diet quality and levels of overweight and obesity in Australia^(15,16)^.

Food purchase decisions are influenced by multiple factors such as food characteristics (sensory, perceptual), marketing (including price, promotion, etc.), environmental factors (social and physical environments), personal-state factors (biological needs, moods, etc.), cognitive factors (information, knowledge, attitude, etc.) and socio-cultural factors (culture, religion, etc.)^(17)^. A natural experiment, such as the one conducted in this study, is not designed to control for all such factors and, consequently, cannot be used to establish a causal relationship. Instead, our analysis approach was designed to identify the association between HSR adoption and the changes in actual consumer purchase behaviour, separate from any other general trends in purchase behaviour. The observed patterns in the healthiness of purchases and the adoption of HSR across categories and over time in our study are consistent with a positive association between the healthiness of purchases and HSR labelling.

Our results are consistent with the findings in the existing literature that food labelling, in general, increases the healthiness of consumer food purchases, and are therefore likely to be beneficial from a public health perspective^(9–11,13,18,19)^. However, there are also some exceptions, such as the study on the effect of HSR on household food purchases conducted in New Zealand, which found a null effect^(12)^. Existing studies on nutrition summary indicators are limited in scope to few product categories across a few grocery stores and therefore do not account for the implications of households’ switching behaviour across products within a category, across product categories and grocery stores^(9–11)^. This large-scale empirical study is the first to analyse the changes in the total packaged food purchases of households before and after the introduction of HSR.

However, this study is not without limitations. First, the study was only able to examine sales over a time period in which adoption of HSR was relatively low (from 0 % adoption in 2014 to adoption on 35 % of packaged products in 2018). It will be important to examine the impact of HSR when a higher proportion of products display the labels. This will offer further insights into the extent to which mandating HSR labels can be expected to improve the healthiness of food purchases. It will also be interesting to see the extent to which the observed changes in healthiness are sustained over time.

Second, we did not examine the changes in the impact of HSR on the healthiness of product formulation. In this regard, a number of studies have identified that HSR can lead to product reformulation and new-product development that is healthier^(4,5,12,20)^. Future research can also analyse the extent to which any observed changes in household-level basket healthiness are due to changes in the mix of household purchases as opposed to changes in product formulation. In addition, nutrition data in the FoodSwitch database were updated annually, and the manual collection of nutrition data was undertaken at the end of the study period, and therefore, the nutrition data in the study did not take any potential product formulation and labelling changes in the intervening time periods into account.

Third, datasets from syndicated data providers such as Nielsen could have potential selection biases. Specifically, panel members could differ from the general population in terms of unobservables (e.g. interest in food). To assess the extent to which Nielsen Homescan data are representative of Australian population, we undertook a comparison of the socio-demographics of the Nielsen panel with those of the general population (see online supplementary material, Supplemental Appendix 4). We found that Nielsen panel represents different demographic groups adequately, but the share of various demographic groups did not correspond precisely with the broader population. In this regard, we conducted stratified analysis by demographic sub-groups (see online supplementary material, Supplemental Appendix 2) to demonstrate that our results are consistent for different sub-groups. Further, studies that have investigated the selection bias and composition differences between syndicated data sources such as Nielsen and the broader population have found that the overall accuracy of self-reported data by Homescan panellists was comparable to those from widely used public surveys such as consumer expenditure surveys^(21,22)^. Nevertheless, the role of self-selection bias in influencing our results cannot be completely ruled out. In particular, to the extent that households who select to participate in the Nielsen Homescan panel tend to differ from those in the broader population in terms of unobserved attributes (e.g. interest in food), and the extent to which these attributes influence their responses to the HSR scheme, our estimates could differ from the true population estimates.

Fourth, to avoid biases in our estimates due to household attrition in the panel, we have included only those households that remained throughout the study period for our main analysis. We also assessed the robustness of our findings to an extended sample of households that remained in the sample for at least 2 years. Nevertheless, by not including households that were not available for the entire study period, our analysis sample may not adequately represent some of the population sub-groups that may be more likely to exhibit attrition bias (e.g. lower socio-economic groups). In addition, the Nielsen dataset does not allow us to identify potential under-reporting of purchases by households. Any systematic under-reporting behaviour by panellists households could potentially influence our model estimates.

Further, we only analysed purchasing data and do not have consumption data and so the study could not directly estimate changes in consumption and resultant changes in diet quality. However, we know that purchases are strongly correlated with consumption, particularly for packaged food where spoilage/wastage is likely to be lower than fresh food^(23,24)^. In addition, the Nielsen Homescan dataset did not cover grocery purchases in non-traditional channels, such as ethnic stores and bakeries etc. and out-of-home food purchases (e.g. fast food), and therefore, our analysis did not account for the implications of HSR for purchases through these channels. Also, given that the outcome variables in our analyses are the healthiness of purchases at aggregate household basket level, and price indices are also at aggregate level, we did not include detailed analysis of price changes. Examining the effect of price changes on consumers’ switching behaviour across products with different HSR and the net effect of these changes warrants a more granular analysis and is an important direction for future research.

The findings of the study, based on the actual purchase behaviour of a large number of households over a long period, indicate that, once a substantial number of packaged food products adopted the voluntary HSR labels, there was an increasing trend in the healthiness of household packaged food purchases. While the magnitude of the observed changes in the healthiness was relatively small, previous modelling studies indicate that the impact of changes of this magnitude on the healthiness of population diets and health outcomes is likely to be substantial^(25)^. When coupled with other evidence showing that the HSR is easily understood by users^(26)^, including by diverse socio-economic groups^(27)^, and is likely to drive improvements in the healthiness of the nutrient composition of packaged food^(17,28)^, this study provides strong support for widespread adoption of HSR on packaged food from a population health perspective. Given that current voluntary regulation of HSR has led to less than 50 % implementation over more than 8 years^(29)^, mandatory regulations are likely to be required to ensure comprehensive adoption.

There is a general agreement that government-led policies and regulations, such as interpretive front-of-pack nutrition labelling, are needed to address high rates of obesity^(30)^. Nutrition labelling has also been widely noted as a key policy option for promoting healthy diets, including in the WHO Global Action Plan for the Prevention and Control of Noncommunicable Diseases 2013–2020^(31)^. However, lack of robust evidence about the effectiveness of interpretive front-of-pack nutrition labelling on consumer purchases has often been cited as a barrier to their widespread, mandatory implementation^(32)^. Our study contributes towards building an evidence base that demonstrates the effectiveness of nutrition summary indicators in promoting healthier diets.

## Supporting information

Seenivasan et al. supplementary material 1 Seenivasan et al. supplementary material

## Supplementary material

For supplementary material accompanying this paper visit https://doi.org/10.1017/S1368980024000892
