# Supplementary material for: Trends (2014–2018) in the healthiness of packaged food purchases of Australian consumers before and after the introduction of voluntary Health Star Rating nutrition labels
Source: Public Health Nutr. 2024 Apr 11;27(1):e144. doi: 10.1017/S1368980024000892 (PMC11617420; doi:10.1017/S1368980024000892)
Supplement: Seenivasan et al. supplementary material 1 — Seenivasan et al. supplementary material [file S1368980024000892sup001.docx]

**Trends (2014-2018) in the healthiness of packaged food purchases of Australian consumers before and after the introduction of voluntary Health Star Rating nutrition labels**

**Supplementary appendix**

**1. Econometric methods**

We first analysed the changes in the food basket healthiness before and after the introduction of HSR. As HSR is a voluntary system, its adoption by products overtime exhibited a non-linear trend. For example, 9.8% of products available in the Foodswitch database had adopted HSR by 2015, 15.9% of products by 2016 and 35.6% of products by 2018. Given the non-linear trend in the uptake of HSR, we used two different approaches to account for its potential non-linear association with food basket healthiness.

First, we estimated the quarterly changes in the food basket healthiness within households using a regression model (quarter-wise fixed effects model) with household and quarter fixed effects as specified in equation (1).

$Food\_{basket\_healthiness}_{ht}=\alpha_{h}+\beta_{t}+\varepsilon_{ht}$ (1)

Here,$Food\_{basket\_healthiness}_{ht}$ is the healthiness of packaged food purchases of household *h* in quarter *t*. In equation (1), household fixed effects ($\alpha_{h})$ control for the differences in basket healthiness across households due to differences in their observed (e.g. demographics) and unobserved (e.g. household preferences) household characteristics. Quarter fixed effects ($\beta_{t})$estimate the average changes in food basket healthiness within households relative to the first quarter. This model allows us to analyse within-households trends in the healthiness of purchases during the study period (2014-2018). In addition to the full sample, we also analysed the trends in the basket HSR of households by sub-groups based on age (<44 years, 45-54 years, > 55 years), gender (male/female) and household income (<$40000, $40-$80000, $80-$140,000, > $140,000), by estimating the quarter-wise fixed effects model separately for each sub-group.

Second, we estimated a regression model (quadratic trend model) with household fixed effects and quadratic time trend as specified in equation (2). In this model, we also included controls for prices of products and seasonality.

$$Food\_{basket\_healthiness}_{ht}=\alpha_{h}+\beta_{1}{Time\_trend}_{t}+\beta_{2} {Time\_trend}_{t}^{2}+\beta_{3}{Price\_index\_HSR1}_{t}+\beta_{4}{Price\_index\_HSR2}_{t}+\beta_{5}{Price\_index\_HSR3}_{t}+\beta_{6}{Price\_index\_HSR4}_{t}+\beta_{7}{Price\_index\_HSR5}_{t}+\beta_{8} {Summer\_qtr}_{t}+\beta_{9}{Autumn\_qtr}_{t}{+\beta_{10}{Winter\_qtr}_{t}+ \varepsilon}_{ht}$$

 (2)

Here, $Time\_trend$ refers to the linear quarterly time trend common across all households. To control for prices, we included five quarterly price indices - one each for products whose HSR are within the following ranges: 0.5-1, 1.5-2, 2.5-3, 3.5-4 and 4.5-5. Specifically, we calculated the share weighted average price per serve of products whose HSR are within the above ranges. We used the dollar shares of a product in the total dollar sales of all products in that HSR range as the weight. These price indexes control for any differential changes in prices of products with different HSR ratings over time. To control for seasaonlity, we included dummy variables to represent quarters that correspond to summer, winter and autumn seasons (relative to spring).

In the third and fourth model specifications, we examined whether the observed trends in the healthiness of households’ packaged food purchases are consistent with households’ purchasing healthier foods after HSR labelling. Specifically, our third model specification (in equation 3) estimated the association between the food basket healthiness and the purchases of HSR-labelled products by household. This analysis utilised the longitudinal (over time) variation in the purchases of HSR-labelled products by households as well as the cross-sectional variation across households in the extent of purchases of HSR-labelled products to test whether the healthiness of the food basket is higher for households that purchased more HSR-labelled products. If HSR labelling doesn’t lead to improvement in the healthiness of household purchases (i.e. food basket healthiness), then there wouldn’t be any significant positive association between the food basket healthiness measures (which would remain unchanged) and the proportion of HSR labelled products in the basket (which would exhibit an increasing trend as more products display label over time). Any positive association between the proportion of HSR labelled products in the basket and the basket healthiness on the other hand would be consistent with households purchasing healthier products after HSR labelling.

$$Food\_{basket\_healthiness}_{ht}=\alpha_{h}+\beta_{1} {Time\_trend}_{t}+\beta_{2}{\%\_HSR\_labelled\_products}_{ht}+\beta_{3}{Price\_index\_HSR1}_{t}+\beta_{4}{Price\_index\_HSR2}_{t}+\beta_{5}{Price\_index\_HSR3}_{t}+\beta_{6}{Price\_index\_HSR4}_{t}+\beta_{7}{Price\_index\_HSR5}_{t}+\beta_{8} {Summer\_qtr}_{t}+\beta_{9}{Autumn\_qtr}_{t}{+\beta_{10}{Winter\_qtr}_{t}+ \varepsilon}_{ht}$$

 (3)

Here,${\%\_HSR\_labelled products}_{ht}$ is the proportion of HSR-labelled products in household h’s basket in quarter t. $\beta_{1}$ captures the effect of linear time trend and $\beta_{2}$ captures the association between the proportion of the HSR-labelled products in the food basket and the healthiness of the basket.

The proportion of HSR labelled products in the basket captures what proportion of products actually displayed HSR, and is calculated from the Foodswitch database. Besides the nutrition information, the FoodSwitch database also captured whether a product displayed HSR or not. We use this information to calculate the proportion of products in the basket that actually displayed HSR labels. We also control for price indices and seasonality in the analysis.

In the fourth model, we tested whether household purchases were healthier in categories where more products were HSR-labelled, using a multi-level fixed effects difference-in-differences type regression model. Specifically, the model (in equation 4) estimated the change in the healthiness of households’ category-specific purchases before and after the introduction of HSR labels relative to control categories where HSR labels were not adopted, and how it varied with the proportion of products in categories that were HSR-labelled.

${Category\_healthiness}_{hct}=\alpha_{h}+\gamma_{c}+⍴ {Time\_trend}_{t}+ \beta_{1}{Post\_HSR}_{ct}+\beta_{2}{Proportion\_HSR\_products}_{ct}++\beta_{3} {Summer\_qtr}_{t}+\beta_{4}{Autumn\_qtr}_{t}+\beta_{5}{Winter\_qtr}_{t}+\varepsilon_{hct}$ (4)

Here, ${Category\_healthiness}_{hct}$ is the HSR of category-specific purchases of household *h* in category *c* in quarter *t*. $\alpha_{h}$ and $\gamma_{c}$ are household and category specific fixed effects, and $⍴$ captures the effect of linear time trend. $Post\_{HSR}_{ct}$ is a categorical variable which indicates whether HSR label was adopted by products in a category c in quarter t. Conditional on $Post\_{HSR}_{ct}$ being 1, the proportion of HSR-labelled products in category c in time t (${Proportion\_HSR\_products}_{ct})$ will be greater than 0. The coefficient of this term captured how the effect of HSR adoption on the healthiness of household purchases varied depending on the proportion of products in the category that were HSR-labelled. This analysis utilised both longitudinal as well as cross-sectional variation across categories in the proportion of HSR-labelled products.

We used household purchases in all studied 114 categories for this analysis. The control group for this analysis included categories where HSR labels weren’t adopted during the study period (e.g. ethnic foods, mixes and batters, meat and fish pastes etc.). Additionally, categories where HSR adoption started in later years served as control groups for categories where HSR adoption started earlier. Specifically, after its endorsement in 2014, HSR label was adopted by products starting from 2014 in some categories (e.g. breakfast cereals), whereas they

weren’t adopted in some categories (e.g. eggs) till 2016 and some other categories (e.g. canned meals, carbonated fruit juice, canned hams/franks & hot dogs, pate) till 2017. The proportion of HSR adoption within categories also increased over time. For example, in the breakfast cereals category, the proportion of HSR-labelled products increased from 50% in 2015 to over 70% in 2018. Categories where HSR labels adoption started in later years (e.g. 2017) served as additional control groups for categories where HSR adoption started earlier (e.g. 2014) for the intervening years (e.g. 2015 and 2016). We also controlled for seasonality in this analysis. However, due to the unavailability of products in all HSR ranges in all studied categories, price indices couldn’t be constructed at category level.

**2. Sub-group analysis of the healthiness trends**

We also analysed the trends in the food basket HSR of households by sub-groups based on age (<44 years, 45-54 years, > 55 years) and gender (male/female) of the primary shopper, as well as the household income (<$40000, $40-$80000, $80-$140,000, > $140,000). We consistently found a similar U-shaped trend for the food basket HSR and an inverted-U shaped trend for the total energy in the food basket for all sub-groups as shown in Supplementary Figures 1a-c (corresponding estimates are shown in Supplementary Tables 8-10).

Specifically, in terms of gender, there were no significant differences in the trends in basket HSR between households with male and female primary shoppers. In terms of age groups, the basket HSR of households with older primary shoppers (greater than 55 years of age) exhibited relatively more negative trend than their counterparts. In terms of income levels, the basket HSR of households with lower incomes (<$40,000) exhibited a relatively more negative trend than their counterparts. However, the differences across the sub-groups were very small in magnitude and predominantly insignificant.

**Supplementary Figure 1a. Trends in the food basket Health Star Ratings by gender**

**Supplementary Figure 1b. Trends in the food basket Health Star Ratings by age group**

**Supplementary Figure 1c. Trends in the food basket Health Star Ratings by income group**

**3. Robustness checks by including fresh food categories**

As HSR was applicable only for packaged foods at the time of the study and the nutrition information wasn’t available for fresh food categories (fruits, vegetables etc.), our main analysis focused only on packaged food categories. To assess the robustness of our results, we also included fresh foods in the calculation of basket HSR. Specifically, we assumed the HSR of all fresh food products to be 5, as per the recent guidelines on HSR^[[1]](#footnote-1)^. We then calculated the dollar share weighted average basket HSR for all food products (fresh and packaged) purchased by households. Results of this anlaysis, shown in supplementary figure 2 (estimates presented in supplementary table 7) show a similar U-shaped trend in basket HSR, consistent with our findings from the main analysis excluding fresh foods.

**Supplementary Figure 2. Trends in the the food basket Health Star Ratings (including fresh food categories)**

**4. Representativeness of the Nielsen Homescan panel data**

We undertook a comparison of the demographic composition of the Nielsen Homescan panel dataset with that of overall Australian population to examine the representation of different demographic sub-groups in the Nielsen dataset. However, as the Nielsen panel has household-level demographics (as opposed to individual-level demographics in most population datasets), and the definitions of various demographic sub-groups in the Nielsen dataset don’t correspond one to one with those in the population surveys, we have mapped Nielsen panel sub-groups with the closest population groups in supplementary table 1.

As shown in supplementary table 1, Nielsen panel represents different demographic groups adequately, but the share of demographic groups in the Nielsen panel doesn’t correspond exactly to that of the broader population. In this regard, we have conducted stratified analysis by various demographic sub-groups, and our results are generally consistent across various groups (supplementary appendix 2), indicating that our findings are not specific to a sub-group. Further, we have estimated all our models with only those households that have remained in the panel for all five years to mitigate any effects due to changes in panel composition over time.

**Supplementary Table 1: Demographic Profiles of Households in the Nielsen Panel Dataset**

| **Demographic characteristics** | **% of households in the Nielsen Homescan sample** | **% of households/individuals in the population^1^** |
| --- | --- | --- |
| **Gender of primary shopper** | | |
| Male | 23.26% | 49.3% |
| Female | 76.73% | 50.7% |
| **Household income** | | |
| 0 - $40,000 | 28.96% | 21.31% |
| $40,000 - $80,000 | 29.89% | 21.81% |
| $80,000 - $140,000 | 28.83% | 32.16% |
| > $140,000 | 12.33% | 24.73% |
| **Age of the primary shopper** | | |
| Less than 35 | 4% | 32.4% |
| 35-44 | 17.20% | 17% |
| 45-54 | 25.24% | 15.42% |
| 55 + | 53.56% | 35.13% |
| **Household size** | | |
| 1 | 21.94% |  |
| 2 | 33.63% |  |
| 3 | 16.76% |  |
| 4 | 16.48% |  |
| 5 or more | 11.19% |  |

**^1^**Population data Sources: <https://www.abs.gov.au/articles/new-census-insights-income-australia-using-administrative-data>

<https://profile.id.com.au/australia/household-income>

**Supplementary Table 2: HSR adoption by products in the studied categories**

| **Category** | **% of products with HSR in 2018** | **Category** | **% of products with HSR in 2018** | **Category** | **% of products with HSR in 2018** |
| --- | --- | --- | --- | --- | --- |
| Artificial Sweeteners | 3.1% | Frozen Chilled Desserts | 34.7% | Eggs | 13.8% |
| Asian/Japan Cooking Misc. | 0.0% | Frozen Drinks | 0.0% | Flavoured Milk | 47.5% |
| Baked Beans & Spaghetti | 67.8% | Frozen Fish/Seafood | 56.3% | Flour | 23.4% |
| Biscuits | 29.9% | Frozen Fruit | 54.5% | Fresh Chilled Soup | 100.0% |
| Bottled & Canned Sauces | 30.5% | Frozen Meals | 53.1% | Ready Made Custard | 28.6% |
| Bread | 43.8% | Frozen Meat & Poultry | 30.1% | Rice | 39.3% |
| Breadcrumbs/Coating & Stuffing | 23.8% | Frozen Pastry | 56.3% | Salad Dressings | 20.1% |
| Breakfast Cereals | 71.8% | Frozen Pizza | 59.8% | Sauce & Gravy Mixes | 35.6% |
| Butter & Margarine | 22.2% | Frozen Rice | 80.0% | Savoury Spreads | 25.0% |
| Cake Decorations | 34.4% | Frozen Snacks | 42.4% | Shelf Stable Desserts | 11.2% |
| Cakes/Pies & Pasties Fresh | 52.4% | Frozen Vegetables | 80.6% | Snack Foods | 38.4% |
| Canned Beans/Salads | 37.5% | Fruit Juices & Drinks | 44.1% | Soup | 59.0% |
| Canned Corned Meats | 19.4% | Golden Syrup/Treacle/Molasse | 42.9% | Soup Mix & Pulses | 0.0% |
| Canned Fish & Seafood | 41.8% | Herbs & Spices/Curry Pwd/Pep | 7.1% | Other Frozen Pastry | 0.0% |
| Canned Fruit/Fruit Snacks | 59.7% | Honey | 6.6% | Powdered Drinks / Specialty Beverages | 0.0% |
| Canned Hams/Franks & Hot Dogs | 11.1% | Ice Cream | 32.4% | Soy / Cottage Cheese / Specialty Chilled Foods | 0.0% |
| Canned Meals | 37.9% | Ice Cream Cones & Wafers | 53.3% | Unclassified Dry Grocery | 0.0% |
| Canned Vegetables | 50.2% | Indian Foods | 2.6% | Specialty Bakery | 0.0% |
| Carbonated Beverages | 41.0% | Infant Formulas | 0.0% | Specialty Confectionery | 0.0% |
| Carbonated Fruit Juice | 9.1% | Jam & Marmalade | 13.6% | Stocks & Flavourings | 16.7% |
| Cheese | 24.3% | Marinades | 0.0% | Sugar Confectionery | 34.9% |
| Chilled Cream | 42.6% | Meat & Fish Pastes | 0.0% | Sweet Spreads | 14.3% |
| Chilled Meals | 51.0% | Mexican Food | 10.3% | Tea | 2.9% |
| Chilled Meat & Poultry | 50.5% | Milk Additives/Tonic Food Dr | 29.8% | Tomato Juice | 0.0% |
| Chilled Pasta | 38.5% | Milk White Fresh and longlife | 45.9% | Tomato Paste & Puree | 80.0% |
| Chilled Savoury Pastry | 0.0% | Mixes & Batters | 3.6% | Toppings | 38.5% |
| Chilled Seafood | 26.0% | Mustard | 26.3% | Unprocessed & Baking Nuts | 65.5% |
| Chilled Vegetable Protein | 70.1% | New Age Beverages | 47.6% | Vegetable & Yeast Extracts | 0.0% |
| Chocolate Confectionery | 37.2% | Non-Carbonated Bev Cordial S | 36.0% | Vegetable Juice | 0.0% |
| Christmas Confectionery | 30.0% | Non-Carbonated Mineral Water | 33.3% | Vinegar | 14.9% |
| Cocoa & Cooking Chocolate | 49.1% | Oils & Fats | 28.0% | Whole Pickles | 0.0% |
| Coconut | 50.0% | Packaged & Prepared Meals | 46.0% | Wrapped Health Snacks | 37.7% |
| Coconut Cream & Milk | 19.0% | Pasta/Noodles | 22.4% | Yogurt & Dairy Dessert | 20.6% |
| Coffee | 18.4% | Pastry Sheets | 0.0% | Yogurt Drinks | 0.0% |
| Coffee Substitutes | 0.0% | Pate | 13.0% |  |  |
| Cooking Wine | 0.0% | Peanut Butter | 45.4% |  |  |
| Dr Ck/Pudding/Cheesecake Mixes | 18.5% | Pickles & Relishes | 20.9% |  |  |
| Dried Fruit | 39.7% | Prepacked Smallgoods | 14.7% |  |  |
| Dried Vegetables | 23.1% | Prepared Dips | 15.1% |  |  |
| Easter Confectionery | 0.0% | Processed Milk Products | 32.4% |  |  |

**Supplementary Table 3: Trends in Basket Healthiness (Estimates from the quarter-wise fixed effects model)**

| **Variable** | **Food basket HSR (Dollar share weighting)** | **Food basket HSR (Energy share weighting)** | **Total energy (KJ) in the basket** |
| --- | --- | --- | --- |
| **Q2, 2014** | -0.031 (-0.037 to -0.024) ^*^ | -0.037 ( -0.044 to -0.030) ^*^ | 0.075 (0.057 to 0.093) ^*^ |
| **Q3, 2014** | -0.024 (-0.030 to -0.017) ^*^ | -0.037 ( -0.044 to -0.030) ^*^ | 0.128 (0.110 to 0.146) ^*^ |
| **Q4, 2014** | -0.086 (-0.093 to -0.079) ^*^ | -0.108 ( -0.115 to -0.101) ^*^ | 0.156 (0.138 to 0.174) ^*^ |
| **Q1, 2015** | -0.025 (-0.032 to -0.018) ^*^ | -0.030 ( -0.037 to -0.018) ^*^ | 0.093 (0.075 to 0.111) ^*^ |
| **Q2, 2015** | -0.045 (-0.051 to -0.038) ^*^ | -0.046 ( -0.053 to -0.023) ^*^ | 0.155 (0.137 to 0.173) ^*^ |
| **Q3, 2015** | -0.044 (-0.051 to -0.037) ^*^ | -0.054 ( -0.061 to -0.047) ^*^ | 0.175 (0.157 to 0.193) ^*^ |
| **Q4, 2015** | -0.094 (-0.100 to -0.087) ^*^ | -0.115 ( -0.122 to -0.108) ^*^ | 0.190 (0.172 to 0.208) ^*^ |
| **Q1, 2016** | -0.043 (-0.049 to -0.036) ^*^ | -0.040 ( -0.047 to -0.033) ^*^ | 0.110 (0.092 to 0.128) ^*^ |
| **Q2, 2016** | -0.027 (-0.034 to -0.021) ^*^ | -0.040 ( -0.047 to -0.033) ^*^ | 0.193 (0.175 to 0.211) ^*^ |
| **Q3, 2016** | -0.051 (-0.058 to -0.044) ^*^ | -0.069 ( -0.077 to -0.062) ^*^ | 0.203 (0.185 to 0.221) ^*^ |
| **Q4, 2016** | -0.111 (-0.117 to -0.104) ^*^ | -0.138 ( -0.145 to -0.131) ^*^ | 0.225 (0.207 to 0.243) ^*^ |
| **Q1, 2017** | -0.033 (-0.040 to -0.027) ^*^ | -0.039 ( -0.046 to -0.032) ^*^ | 0.108 (0.090 to 0.126) ^*^ |
| **Q2, 2017** | -0.064 (-0.071 to -0.057) ^*^ | -0.064 ( -0.071 to -0.057) ^*^ | 0.157 (0.139 to 0.175) ^*^ |
| **Q3, 2017** | -0.057 (-0.063 to -0.050) ^*^ | -0.067 ( -0.074 to -0.060) ^*^ | 0.179 (0.161 to 0.197) ^*^ |
| **Q4, 2017** | -0.108 (-0.115 to -0.101) ^*^ | -0.129 ( -0.136 to -0.122) ^*^ | 0.173 (0.155 to 0.191) ^*^ |
| **Q1, 2018** | -0.033 (-0.040 to -0.027) ^*^ | -0.036 ( -0.043 to -0.029) ^*^ | 0.085 (0.067 to 0.103) ^*^ |
| **Q2, 2018** | -0.022 (-0.029 to -0.016) ^*^ | -0.039 ( -0.046 to -0.032) ^*^ | 0.112 (0.094 to 0.130) ^*^ |
| **Q3, 2018** | -0.038 (-0.045 to -0.032)^*^ | -0.069 ( -0.076 to -0.062)^*^ | 0.131 (0.113 to 0.149) ^*^ |
| **Q4, 2018** | -0.096 (-0.102 to -0.089) ^*^ | -0.140 ( -0.147 to -0.133) ^*^ | 0.088 (0.070 to 0.106) ^*^ |
| **R^2^** | 0.619 | 0.643 | 0.565 |

Note: Household specific fixed effects are not shown in the above table.

^*^Significant at 95% Confidence Interval

**Supplementary Table 4: Trends in Food Basket Healthiness (Estimates from the Quadratic trend model)**

| **Variable** | **Food basket HSR (Dollar share weighting)** | **Food basket HSR (Energy share weighting)** | **Total energy (KJ) in the food basket** |
| --- | --- | --- | --- |
| **Time_trend** | -0.0067  (-0.0087 to -0.0048)^*^ | -0.0074  (-0.0094 to -0.0053)^*^ | 0.0270  (0.0218 to 0.0323)^*^ |
| **Time_trend^2^** | 0.0002  (0.0001 to 0.0003)^*^ | 0.0002  (0.0001 to 0.0003)^*^ | -0.0014  (-0.0016 to -0.0012)^*^ |
| **Price_HSR_1** | -0.2175  (-0.3046 to -0.1305)^*^ | -0.1339  (-0.2259 to -0.0420)^*^ | -0.2511  (-0.4861 to -0.0161)^*^ |
| **Price_HSR_2** | 0.0101  (-0.0277 to 0.0478) | -0.0953  (-0.1352 to -0.0555)^*^ | -0.0604  (-0.1623 to 0.0414) |
| **Price_HSR_3** | -0.1490  (-0.3521 to 0.0542) | -0.0438  (-0.2584 to 0.1707) | 0.4380  (-0.1103 to 0.9863) |
| **Price_HSR_4** | 0.1292  (0.0290 to 0.2293)^*^ | 0.1187  (0.0130 to 0.2245)^*^ | 0.5150  (0.2448 to 0.7853)^*^ |
| **Price_HSR_5** | 0.0800  (0.0450 to 0.1150)^*^ | 0.0199  (-0.0171 to 0.0568) | 0.0390  (-0.0555 to 0.1334) |
| **Summer** | 0.0713  (0.0651 to 0.0775)^*^ | 0.0924  (0.0858 to 0.0989)^*^ | -0.0662  (-0.0829 to -0.0494)^*^ |
| **Winter** | 0.0572  (0.0531 to 0.0613)^*^ | 0.0623  (0.0580 to 0.0666)^*^ | 0.0011  (-0.0100 to 0.0122) |
| **Autumn** | 0.0565  (0.0524 to 0.0606)^*^ | 0.0755  (0.0711 to 0.0798)^*^ | -0.0234  (-0.0345 to -0.0123)^*^ |
| **R^2^** | 0.619 | 0.642 | 0.565 |

Note: Household specific fixed effects are not shown in the above table.

^*^Significant at 95% Confidence Interval

**Supplementary Table 5: Trends in Basket Healthiness - Robustness check with extended sample (Quarter-wise fixed effects model)**

| **Variable** | **Food basket HSR (Dollar share weighting)** | **Food basket HSR (Energy share weighting)** | **Total energy (KJ) in the basket** |
| --- | --- | --- | --- |
| **Q2, 2014** | -0.028 ( -0.034 to -0.022) ^*^ | -0.034 ( -0.041 to -0.028) ^*^ | 0.083 (0.058 to 0.107) ^*^ |
| **Q3, 2014** | -0.022 ( -0.028 to -0.015) ^*^ | -0.034 ( -0.040 to -0.027) ^*^ | 0.145 (0.121 to 0.169) ^*^ |
| **Q4, 2014** | -0.082 ( -0.088 to -0.076) ^*^ | -0.104 ( -0.111 to -0.097) ^*^ | 0.170 (0.146 to 0.194) ^*^ |
| **Q1, 2015** | -0.024 ( -0.031 to -0.018) ^*^ | -0.028 ( -0.035 to -0.022) ^*^ | 0.106 (0.082 to 0.130) ^*^ |
| **Q2, 2015** | -0.042 ( -0.048 to -0.036) ^*^ | -0.044 ( -0.051 to -0.038) ^*^ | 0.158 (0.134 to 0.182) ^*^ |
| **Q3, 2015** | -0.043 ( -0.049 to -0.036) ^*^ | -0.051 ( -0.058 to -0.045) ^*^ | 0.168 (0.144 to 0.192) ^*^ |
| **Q4, 2015** | -0.090 ( -0.096 to -0.084) ^*^ | -0.110 ( -0.117 to -0.104) ^*^ | 0.164 (0.140 to 0.188) ^*^ |
| **Q1, 2016** | -0.039 ( -0.045 to -0.032) ^*^ | -0.037 ( -0.043 to -0.030) ^*^ | 0.095 (0.070 to 0.119) ^*^ |
| **Q2, 2016** | -0.023 ( -0.029 to -0.017) ^*^ | -0.035 ( -0.042 to -0.028) ^*^ | 0.173 (0.149 to 0.198) ^*^ |
| **Q3, 2016** | -0.048 ( -0.054 to -0.041) ^*^ | -0.067 ( -0.073 to -0.060) ^*^ | 0.195 (0.170 to 0.219) ^*^ |
| **Q4, 2016** | -0.105 ( -0.111 to -0.098) ^*^ | -0.134 ( -0.140 to -0.127) ^*^ | 0.206 (0.181 to 0.230) ^*^ |
| **Q1, 2017** | -0.031 ( -0.037 to -0.025) ^*^ | -0.038 ( -0.044 to -0.031) ^*^ | 0.089 (0.064 to 0.114) ^*^ |
| **Q2, 2017** | -0.062 ( -0.069 to -0.056) ^*^ | -0.062 ( -0.068 to -0.055) ^*^ | 0.156 (0.132 to 0.181) ^*^ |
| **Q3, 2017** | -0.054 ( -0.061 to -0.048) ^*^ | -0.063 ( -0.070 to -0.056) ^*^ | 0.188 (0.164 to 0.213) ^*^ |
| **Q4, 2017** | -0.105 ( -0.112 to -0.099) ^*^ | -0.125 ( -0.132 to -0.118) ^*^ | 0.160 (0.135 to 0.185) ^*^ |
| **Q1, 2018** | -0.030 ( -0.037 to -0.024) ^*^ | -0.034 ( -0.041 to -0.028) ^*^ | 0.073 (0.048 to 0.098) ^*^ |
| **Q2, 2018** | -0.018 ( -0.025 to -0.011) ^*^ | -0.033 ( -0.040 to -0.026) ^*^ | 0.099 (0.073 to 0.124) ^*^ |
| **Q3, 2018** | -0.033 ( -0.040 to -0.026)^*^ | -0.064 ( -0.071 to -0.057)^*^ | 0.124 (0.099 to 0.150) ^*^ |
| **Q4, 2018** | -0.092 ( -0.099 to -0.085) ^*^ | -0.136 ( -0.143 to -0.129) ^*^ | 0.089 (0.064 to 0.115) ^*^ |
| **R^2^** | 0.598 | 0.623 | 0.623 |

Note: Household specific fixed effects are not shown in the above table.

^*^Significant at 95% Confidence Interval

**Supplementary Table 6: Trends in Basket Healthiness (Quadratic trend model) - Robustness check with extended sample**

| **Variable** | **Food basket HSR (Dollar share weighting)** | **Food basket HSR (Energy share weighting)** | **Total energy (KJ) in the food basket** |
| --- | --- | --- | --- |
| **Time_trend** | -0.0056  (-0.0075 to -0.0037)^*^ | -0.0068  (-0.0087 to -0.0048)^*^ | 0.0236  (0.0164 to 0.0307)^*^ |
| **Time_trend^2^** | 0.0002  (0.0001 to 0.0002)^*^ | 0.0002  (0.0001 to 0.0003)^*^ | -0.0012  (-0.0015 to -0.0009)^*^ |
| **Price_HSR_1** | -0.2106  (-0.2934 to -0.1279)^*^ | -0.1259  (-0.2132 to -0.0385)^*^ | 0.0012  (-0.3165 to 0.3190) |
| **Price_HSR_2** | 0.0209  (-0.0163 to 0.0581) | -0.0924  (-0.1316 to -0.0531)^*^ | -0.0590  (-0.2017 to 0.0838) |
| **Price_HSR_3** | -0.1094  (-0.3075 to 0.0887) | -0.0110  (-0.2202 to 0.1982) | 0.7008  (-0.0600 to 1.4617) |
| **Price_HSR_4** | 0.1752  (0.0761 to 0.2743)^*^ | 0.1476  (0.0430 to 0.2522)^*^ | 0.5477  (0.1642 to 0.9252)^*^ |
| **Price_HSR_5** | 0.0891  (0.0552 to 0.1230)^*^ | 0.0209  (-0.0149 to 0.0567) | -0.0552  (-0.1854 to 0.0751) |
| **Summer** | 0.0715  (0.0655 to 0.0775)^*^ | 0.0910  (0.0846 to 0.0973)^*^ | -0.0647  (-0.0878 to -0.0415)^*^ |
| **Winter** | 0.0586  (0.0545 to 0.0626)^*^ | 0.0631  (0.0588 to 0.0674)^*^ | 0.0117  (-0.0039 to 0.0273) |
| **Autumn** | 0.0569  (0.0529 to 0.0609)^*^ | 0.0755  (0.0712 to 0.0797)^*^ | -0.0131  (-0.0286 to 0.0023) |
| **R^2^** | 0.598 | 0.622 | 0.623 |

Note: Household specific fixed effects are not shown in the above table.

^*^Significant at 95% Confidence Interval

**Supplementary Table 7: Trends in Basket Healthiness - Robustness check including fresh foods**

| **Variable** | **Food basket HSR [95% CI]** |
| --- | --- |
| **Time_trend** | -0·0010^***^  [-0·0018 to -0·0002] |
| **Time_trend^2^** | 0·0001^***^  [0·0001 to 0·0002] |
| **Summer** | 0·0779^***^  [0·0747 to 0·0811] |
| **Winter** | 0·0189^***^  [0·0158 to 0·0221] |
| **Autumn** | 0·0156^***^  [0·0125 to 0·0188] |
| **R^2^** | 0·704 |

Note: Household specific fixed effects are not shown in the above table.

^***^P < 0·001,^**^P < 0·01, ^*^P < 0·05.

**Supplementary Table 8: Trends in Basket Healthiness (Food basket HSR) by gender**

| **Variable** | **Male** | **Female** |
| --- | --- | --- |
| **Q2, 2014** | -0.024 ( -0.038 to -0.009) ^*^ | -0.033 ( -0.040 to -0.025) ^*^ |
| **Q3, 2014** | -0.016 ( -0.031 to 0.002) ^*^ | -0.026 ( -0.033 to -0.018) ^*^ |
| **Q4, 2014** | -0.076 ( -0.091 to -0.062) ^*^ | -0.089 ( -0.097 to -0.082) ^*^ |
| **Q1, 2015** | -0.021 ( -0.035 to -0.006) ^*^ | -0.026 ( -0.034 to -0.019) ^*^ |
| **Q2, 2015** | -0.046 ( -0.061 to -0.032) ^*^ | -0.044 ( -0.052 to -0.037) ^*^ |
| **Q3, 2015** | -0.046 ( -0.061 to -0.031) ^*^ | -0.043 ( -0.051 to -0.036) ^*^ |
| **Q4, 2015** | -0.076 ( -0.091 to -0.062) ^*^ | -0.099 ( -0.106 to -0.091) ^*^ |
| **Q1, 2016** | -0.034 ( -0.049 to -0.020) ^*^ | -0.045 ( -0.053 to -0.038) ^*^ |
| **Q2, 2016** | -0.024 ( -0.038 to -0.009) ^*^ | -0.028 ( -0.036 to -0.021) ^*^ |
| **Q3, 2016** | -0.047 ( -0.061 to -0.032) ^*^ | -0.052 ( -0.060 to -0.045) ^*^ |
| **Q4, 2016** | -0.104 ( -0.119 to -0.089) ^*^ | -0.113 ( -0.120 to -0.105) ^*^ |
| **Q1, 2017** | -0.032 ( -0.047 to -0.018) ^*^ | -0.034 ( -0.041 to -0.026) ^*^ |
| **Q2, 2017** | -0.056 ( -0.071 to -0.042) ^*^ | -0.067 ( -0.074 to -0.059) ^*^ |
| **Q3, 2017** | -0.052 ( -0.067 to -0.038) ^*^ | -0.058 ( -0.065 to -0.050) ^*^ |
| **Q4, 2017** | -0.096 ( -0.110 to -0.081) ^*^ | -0.112 ( -0.120 to -0.105) ^*^ |
| **Q1, 2018** | -0.035 ( -0.050 to -0.020) ^*^ | -0.033 ( -0.041 to -0.026) ^*^ |
| **Q2, 2018** | -0.023 ( -0.037 to -0.008) ^*^ | -0.022 ( -0.030 to -0.015) ^*^ |
| **Q3, 2018** | -0.037 ( -0.051 to -0.022) ^*^ | -0.039 ( -0.046 to -0.031) ^*^ |
| **Q4, 2018** | -0.092 ( -0.107 to -0.077) ^*^ | -0.097 ( -0.104 to -0.089) ^*^ |
| **R^2^** | 0.629 | 0.614 |

Note: Household specific fixed effects are not shown in the above table.

^*^Significant at 95% Confidence Interval

**Supplementary Table 9: Trends in Basket Healthiness (Food basket HSR) by age group**

| **Variable** | **<= 44 years** | **45-54 years** | **> 55 years** |
| --- | --- | --- | --- |
| **Q2, 2014** | -0.033 ( -0.051 to -0.014) ^*^ | -0.024 ( -0.032 to -0.011) ^*^ | -0.033 ( -0.041 to -0.025) ^*^ |
| **Q3, 2014** | -0.023 ( -0.042 to -0.004) ^*^ | -0.021 ( -0.032 to -0.007) ^*^ | -0.025 ( -0.034 to -0.017) ^*^ |
| **Q4, 2014** | -0.063 ( -0.082 to -0.044) ^*^ | -0.071 ( -0.079 to -0.058) ^*^ | -0.100 ( -0.109 to -0.092) ^*^ |
| **Q1, 2015** | -0.017 ( -0.035 to 0.002) | -0.020 ( -0.029 to -0.007) ^*^ | -0.030 ( -0.038 to -0.021) ^*^ |
| **Q2, 2015** | -0.039 ( -0.058 to -0.020) ^*^ | -0.032 ( -0.042 to -0.019) ^*^ | -0.052 ( -0.061 to -0.044) ^*^ |
| **Q3, 2015** | -0.034 ( -0.052 to -0.015) ^*^ | -0.042 ( -0.050 to -0.029) ^*^ | -0.048 ( -0.056 to -0.039) ^*^ |
| **Q4, 2015** | -0.068 ( -0.087 to -0.049) ^*^ | -0.075 ( -0.082 to -0.062) ^*^ | -0.110 ( -0.118 to -0.101) ^*^ |
| **Q1, 2016** | -0.030 ( -0.049 to -0.011) ^*^ | -0.036 ( -0.042 to -0.023) ^*^ | -0.050 ( -0.058 to -0.041) ^*^ |
| **Q2, 2016** | -0.021 ( -0.040 to -0.002) ^*^ | -0.017 ( -0.023 to -0.004) ^*^ | -0.033 ( -0.042 to -0.025) ^*^ |
| **Q3, 2016** | -0.045 ( -0.064 to -0.026) ^*^ | -0.045 ( -0.051 to -0.032) ^*^ | -0.056 ( -0.064 to -0.047) ^*^ |
| **Q4, 2016** | -0.086 ( -0.105 to -0.067) ^*^ | -0.097 ( -0.102 to -0.084) ^*^ | -0.124 ( -0.133 to -0.116) ^*^ |
| **Q1, 2017** | -0.012 ( -0.030 to 0.007) | -0.026 ( -0.035 to -0.013) ^*^ | -0.044 ( -0.052 to -0.035) ^*^ |
| **Q2, 2017** | -0.047 ( -0.065 to -0.028) ^*^ | -0.058 ( -0.064 to -0.045) ^*^ | -0.072 ( -0.080 to -0.064) ^*^ |
| **Q3, 2017** | -0.039 ( -0.058 to -0.020) ^*^ | -0.046 ( -0.052 to -0.033) ^*^ | -0.067 ( -0.075 to -0.059) ^*^ |
| **Q4, 2017** | -0.072 ( -0.091 to -0.053) ^*^ | -0.088 ( -0.096 to -0.075) ^*^ | -0.128 ( -0.137 to -0.120) ^*^ |
| **Q1, 2018** | -0.021 ( -0.040 to -0.002) ^*^ | -0.024 ( -0.033 to -0.011) ^*^ | -0.042 ( -0.050 to -0.033) ^*^ |
| **Q2, 2018** | -0.006 ( -0.025 to 0.013) | -0.004 ( -0.011 to 0.009) | -0.035 ( -0.044 to -0.027) ^*^ |
| **Q3, 2018** | -0.037 ( -0.056 to -0.019) ^*^ | -0.021 ( -0.024 to -0.008) ^*^ | -0.046 ( -0.055 to -0.038) ^*^ |
| **Q4, 2018** | -0.071 ( -0.090 to -0.053) ^*^ | -0.074 ( -0.081 to -0.061) ^*^ | -0.113 ( -0.121 to -0.105) ^*^ |
| **R^2^** | 0.529 | 0.609 | 0.655 |

Note: Household specific fixed effects are not shown in the above table.

^*^Significant at 95% Confidence Interval

**Supplementary Table 10: Trends in Basket Healthiness (Food basket HSR) by income group**

| **Variable** | **< $40,000** | **$40,000-$80,000** | **$80,000-$140,000** | **> $140,000** |
| --- | --- | --- | --- | --- |
| **Q2, 2014** | -0.035 ( -0.048 to -0.022) ^*^ | -0.026 ( -0.038 to -0.014) ^*^ | -0.035 ( -0.047 to -0.022) ^*^ | -0.023 ( -0.042 to -0.004) ^*^ |
| **Q3, 2014** | -0.031 ( -0.043 to -0.018) ^*^ | -0.016 ( -0.028 to -0.004) ^*^ | -0.028 ( -0.041 to -0.015) ^*^ | -0.016 ( -0.035 to 0.003) |
| **Q4, 2014** | -0.097 ( -0.110 to -0.085) ^*^ | -0.083 ( -0.095 to -0.071) ^*^ | -0.085 ( -0.098 to -0.073) ^*^ | -0.070 ( -0.089 to -0.051) ^*^ |
| **Q1, 2015** | -0.035 ( -0.048 to -0.023) ^*^ | -0.018 ( -0.030 to -0.006) ^*^ | -0.022 ( -0.034 to -0.009) ^*^ | -0.025 ( -0.044 to -0.006) ^*^ |
| **Q2, 2015** | -0.054 ( -0.066 to -0.041) ^*^ | -0.038 ( -0.050 to -0.026) ^*^ | -0.046 ( -0.059 to -0.034) ^*^ | -0.037 ( -0.056 to -0.018) ^*^ |
| **Q3, 2015** | -0.056 ( -0.069 to -0.043) ^*^ | -0.034 ( -0.046 to -0.022) ^*^ | -0.042 ( -0.054 to -0.029) ^*^ | -0.044 ( -0.063 to -0.025) ^*^ |
| **Q4, 2015** | -0.110 ( -0.123 to -0.098) ^*^ | -0.092 ( -0.104 to -0.080) ^*^ | -0.085 ( -0.098 to -0.073) ^*^ | -0.076 ( -0.095 to -0.057) ^*^ |
| **Q1, 2016** | -0.052 ( -0.065 to -0.040) ^*^ | -0.041 ( -0.053 to -0.029) ^*^ | -0.040 ( -0.053 to -0.028) ^*^ | -0.031 ( -0.050 to -0.011) ^*^ |
| **Q2, 2016** | -0.040 ( -0.053 to -0.028) ^*^ | -0.025 ( -0.037 to -0.013) ^*^ | -0.016 ( -0.028 to 0.003) ^*^ | -0.028 ( -0.047 to -0.009) ^*^ |
| **Q3, 2016** | -0.062 ( -0.075 to -0.049) ^*^ | -0.050 ( -0.062 to -0.038) ^*^ | -0.049 ( -0.062 to -0.036) ^*^ | -0.032 ( -0.051 to -0.013) ^*^ |
| **Q4, 2016** | -0.123 ( -0.136 to -0.111) ^*^ | -0.108 ( -0.120 to -0.096) ^*^ | -0.102 ( -0.115 to -0.090) ^*^ | -0.108 ( -0.127 to -0.089) ^*^ |
| **Q1, 2017** | -0.044 ( -0.056 to -0.031) ^*^ | -0.038 ( -0.050 to -0.026) ^*^ | -0.023 ( -0.035 to -0.010) ^*^ | -0.023 ( -0.042 to -0.004) ^*^ |
| **Q2, 2017** | -0.079 ( -0.092 to -0.067) ^*^ | -0.062 ( -0.074 to -0.050) ^*^ | -0.054 ( -0.066 to -0.038) ^*^ | -0.055 ( -0.075 to -0.036) ^*^ |
| **Q3, 2017** | -0.074 ( -0.086 to -0.061) ^*^ | -0.051 ( -0.063 to -0.039) ^*^ | -0.050 ( -0.063 to -0.035) ^*^ | -0.046 ( -0.065 to -0.027) ^*^ |
| **Q4, 2017** | -0.129 ( -0.142 to -0.116) ^*^ | -0.108 ( -0.120 to -0.096) ^*^ | -0.096 ( -0.108 to -0.082) ^*^ | -0.087 ( -0.106 to -0.068) ^*^ |
| **Q1, 2018** | -0.045 ( -0.058 to -0.032) ^*^ | -0.033 ( -0.045 to -0.021) ^*^ | -0.029 ( -0.042 to -0.015) ^*^ | -0.019 ( -0.038 to 0) |
| **Q2, 2018** | -0.043 ( -0.055 to -0.030) ^*^ | -0.023 ( -0.035 to -0.011) ^*^ | -0.012 ( -0.024 to 0.004) | 0.003 ( -0.016 to 0.022) |
| **Q3, 2018** | -0.057 ( -0.069 to -0.044) ^*^ | -0.039 ( -0.051 to -0.027) ^*^ | -0.030 ( -0.043 to -0.016) ^*^ | -0.013 ( -0.032 to 0.006) |
| **Q4, 2018** | -0.115 ( -0.127 to -0.102) ^*^ | -0.096 ( -0.108 to -0.084) ^*^ | -0.091 ( -0.103 to -0.077) ^*^ | -0.062 ( -0.081 to -0.043) ^*^ |
| **R^2^** | 0.629 | 0.624 | 0.609 | 0.599 |

Note: Household specific fixed effects are not shown in the above table.

^*^Significant at 95% Confidence Interval

1. <http://www.healthstarrating.gov.au/internet/healthstarrating/publishing.nsf/Content/How-to-use-health-stars> [↑](#footnote-ref-1)
